# Deep learning‐based auto‐segmentation of clinical target volumes for radiotherapy treatment of cervical cancer

**DOI:** 10.1002/acm2.13470

**Published:** 2021-11-22

**Authors:** Chen‐Ying Ma, Ju‐Ying Zhou, Xiao‐Ting Xu, Jian Guo, Miao‐Fei Han, Yao‐Zong Gao, Hui Du, Johannes N. Stahl, Jonathan S. Maltz

**Affiliations:** ^1^ Department of Radiation Oncology First Affiliated Hospital of Soochow University Suzhou China; ^2^ Shanghai United Imaging Healthcare, Co. Ltd. Jiading China

**Keywords:** artificial intelligence (AI), auto‐segmentation, cervical cancer, clinical target volume (CTV), deep learning

## Abstract

**Objectives:**

Because radiotherapy is indispensible for treating cervical cancer, it is critical to accurately and efficiently delineate the radiation targets. We evaluated a deep learning (DL)‐based auto‐segmentation algorithm for automatic contouring of clinical target volumes (CTVs) in cervical cancers.

**Methods:**

Computed tomography (CT) datasets from 535 cervical cancers treated with definitive or postoperative radiotherapy were collected. A DL tool based on VB‐Net was developed to delineate CTVs of the pelvic lymph drainage area (dCTV1) and parametrial area (dCTV2) in the definitive radiotherapy group. The training/validation/test number is 157/20/23. CTV of the pelvic lymph drainage area (pCTV1) was delineated in the postoperative radiotherapy group. The training/validation/test number is 272/30/33. Dice similarity coefficient (DSC), mean surface distance (MSD), and Hausdorff distance (HD) were used to evaluate the contouring accuracy. Contouring times were recorded for efficiency comparison.

**Results:**

The mean DSC, MSD, and HD values for our DL‐based tool were 0.88/1.32 mm/21.60 mm for dCTV1, 0.70/2.42 mm/22.44 mm for dCTV2, and 0.86/1.15 mm/20.78 mm for pCTV1. Only minor modifications were needed for 63.5% of auto‐segmentations to meet the clinical requirements. The contouring accuracy of the DL‐based tool was comparable to that of senior radiation oncologists and was superior to that of junior/intermediate radiation oncologists. Additionally, DL assistance improved the performance of junior radiation oncologists for dCTV2 and pCTV1 contouring (mean DSC increases: 0.20 for dCTV2, 0.03 for pCTV1; mean contouring time decrease: 9.8 min for dCTV2, 28.9 min for pCTV1).

**Conclusions:**

DL‐based auto‐segmentation improves CTV contouring accuracy, reduces contouring time, and improves clinical efficiency for treating cervical cancer.

## 1 INTRODUCTION

Cervical cancer is one of the most common malignancies in women worldwide and is second most common after breast cancer. Most cases occur in developing countries, seriously impacting the health of women and representing the leading cause of tumor‐related death in these countries.^1^ Unlike the declining incidence rates owing to the popularization of cervical cancer screening in Western countries, the incidence of cervical cancer in China has continued to rise due to many factors such as sociocultural factors, lack of awareness for physical examinations, medical resource shortages, etc.^2^

Radiotherapy plays a critical role in treating cervical cancer. For early‐stage cervical cancer, radiotherapy is usually administered as a postoperative adjuvant treatment. For locally advanced or metastatic cervical cancer, external beam radiation therapy (EBRT) and chemotherapy followed by brachytherapy (BT) is recommended as the standard treatment modality.^3^ Intensity‐modulated radiation therapy (IMRT) is the most commonly used radiation technique for cervical cancer because it delivers high‐precision therapeutic doses to tumors and reduced doses to organs at risk. To maximize the therapeutic ratio, accurate contouring of the targets and adjacent normal organs is essential in radiotherapy planning for cervical cancer. Accurate segmentation contributes to reducing late toxicities associated with pelvic chemoradiation. This is particularly important because late toxicities such as incontinence, fistulae, and malabsorption may last for many years, causing great harm especially for young patients.^4^, ^5^

Generally, the clinical tumor volume (CTV) for cervical cancer is delineated and confirmed manually by radiation oncologists (ROs) based on gynecological examinations, surgery reports, as well as computed tomography (CT), magnetic resonance imaging (MRI), and other imaging evaluations. The definition of the target volume depends on the doctor's understanding of the clinical guidelines, consensus, and experience.^6^, ^7^, ^8^ There remain inter‐ and intraobserver variations regarding the quality, efficiency, and repeatability of segmentation. Additionally, segmentation of target volumes accounts for the majority of time in radiotherapy planning and is affected by the proficiency of the ROs. In our clinic, target definition typically takes 20–60 min. To overcome these issues, automatic segmentation for radiotherapy planning has become essential. Automatic segmentation has been demonstrated to be effective for improving the consistency of contouring and saving labor.^9^, ^10^

At present, atlas‐based automatic segmentation (ABAS) algorithms are widely used in commercial treatment planning software. However, for organs and tumors that lack clearly defined boundaries or exhibit complex shapes, the results of atlas‐based segmentation are usually unsatisfactory.^11^, ^12^, ^13^ Kim et al.^14^ applied ABAS on patients with endometrial and cervical cancers, the dice similarity coefficient (DSC) and Hausdorff distance (HD) of CTV were 0.79 and 9.7 mm, respectively. Based on the convolutional neural networks (CNNs), artificial intelligence based on deep learning (DL) has been proven to be a promising technology for medical image segmentation. Such DL‐based segmentation algorithms demonstrate significant advantages over classical medical image segmentation methods.^15^, ^16^ Several groups have applied DL to auto‐segment tumor targets that are not amenable to accurate contouring via traditional automatic methods. Lin et al.^17^ constructed and validated a DL contouring tool for auto‐segmenting the primary gross tumor volume (GTV) of nasopharyngeal carcinoma on magnetic resonance (MR) images. The DL‐generated contours demonstrated a high level of accuracy when compared with reference contours (contours reviewed and approved for radiotherapy by senior ROs) in 203 patients (DSC, 0.79; mean surface distance (MSD), 2.0 mm). Furthermore, DL‐based segmentation has been confirmed to improve contouring accuracy, reduce intra‐ and interobserver variation, and shorten contouring time (by 39.4%). Men et al.^18^ applied a deep deconvolutional neural network for segmentation of the primary tumor GTV (GTV‐nx), metastatic lymph node GTV (GTV‐nd), and the CTV of nasopharyngeal carcinoma cases; the resulting DSC values for GTV‐nx, GTV‐nd, and CTV were 80.9%, 62.3%, and 82.6%, respectively, which compared favorably with those obtained by both manual and previously applied automatic methods. Trebeschi et al.^19^ applied DL assistance to the segmentation of rectal cancer on multiparametric MR images and obtained a DSC of 69%.

However, the role of DL‐based tool on auto‐segmentation of CTVs in cervical cancer still remains unexplored. Thus, we investigated the DL‐based tool for CTV contouring of cervical cancer, and compared the accuracy, consistency, and workflow acceleration between the DL‐based auto‐segmentation, DL‐assisted manual contouring, and manual contouring results.

## 2 METHODS

### 2.1 Criteria for data selection and sketch

**FIGURE 1:**
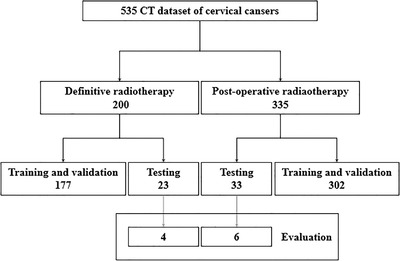
Details of the datasets

CT datasets for 535 cases were collected for cervical cancer patients who received radical or postoperative radiotherapy at the First Affiliated Hospital of Soochow University between January 2013 and June 2019. These data are divided into: (1) dataset 1 consists of 200 patients who received radical radiotherapy and (2) dataset 2 consists of 335 patients who received postoperative adjuvant radiotherapy. Dataset 1 were randomly divided into a training group (*n* = 157), a validation group (*n* = 20), and a testing group (*n* = 23). Dataset 2 were randomly divided into a training group (*n* = 272), a validation group (*n* = 30), and a testing group (*n* = 33). Besides, four patients in dataset 1 and six patients in dataset 2 were randomly selected from both testing groups to generate an evaluation group. All planning CT scans were obtained with a Philips Brilliance Big Bore with slice thickness of 5 mm and field of view of 500 mm. The details of the datasets are presented in Figure 1.

In order to visualize the artery and other blood vessels clearly in CT images, contrast agent is used in all CT scans. The CT scans covered the drainage area of pelvic lymph nodes (3‐mm slices from L3 spine to the middle of femur). The ROs contoured the CTVs on the planning CT images according to guidelines of cervical cancer including Radiation Therapy Oncology Group (RTOG),^20^ Japan Clinical Oncology Group (JCOG),^21^ and Federation International of Gynecology and Obstetrics.^22^ For dataset 1, CTV of the pelvic lymph drainage area (dCTV1) and the parametrial area (dCTV2) were delineated, while CTV of the pelvic lymph drainage area (pCTV1) was delineated in dataset 2. In order to improve data consistency, only the upper third of the vagina was delineated and para‐aortic lymph nodes contourings were omitted. Contours reviewed and approved for radiotherapy by senior ROs were set as reference contours in this study.

### 2.2 Structure of DL network

VB‐Net CNNs are employed at both segmentation phases described above, but each implements a different spatial sampling regime. While the traditional V‐Net algorithm^23^ has achieved good results in many automatic segmentation studies, it often requires training a model that contains a large number of parameters. A V‐Net model file is generally about 250 MB, which not only leads to parameter redundancy, waste of storage space, and reduction of calculation efficiency, but also hinders the promotion and usage of automatic segmentation.

**FIGURE 2:**
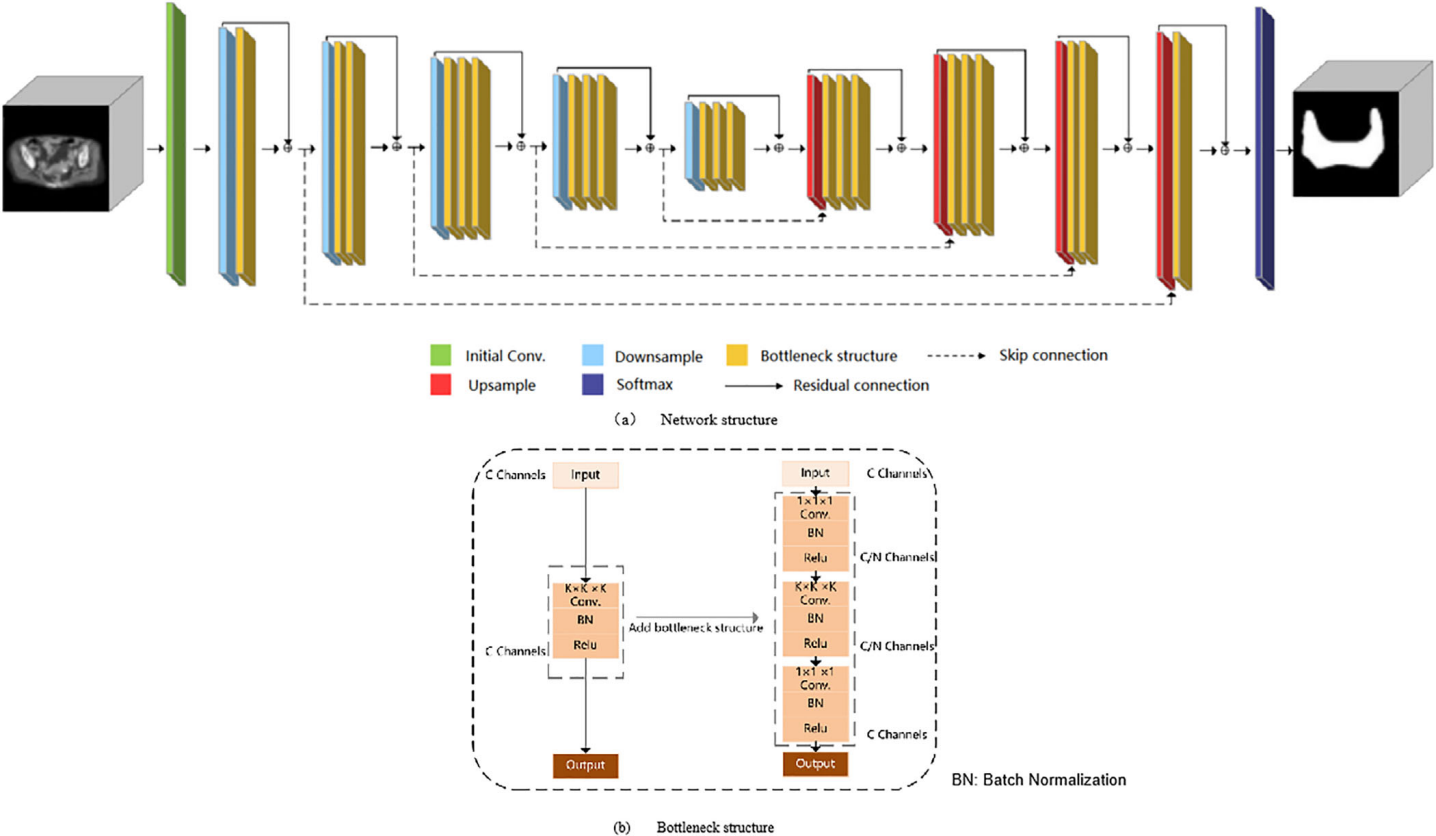
Schematic of the network architecture (a) and flow chart of the bottleneck structure (b)

VB‐Net, a new type of network structure, is proposed as an improvement over V‐Net. The structure of VB‐Net is shown in Figure 2. The residual module in V‐Net was designed using the concept of model compression. The convolution, normalization, and activation layers in V‐Net are replaced by a bottleneck structure, which is the B in VB‐Net. A bottleneck in a neural network is a layer having fewer neurons than its adjacent layers. Such a layer encourages the network to compress feature representations to best fit in the available vector space. The bottleneck structure consists of three convolutional layers. The first and third convolutional layers, which utilize the unit convolution kernel, match the second (bottleneck) convolutional layer with the respective dimensions of the preceding and succeeding layers. The second convolution layer performs spatial convolution on the feature image reduced in dimension by the first convolution layer. Since the spatial convolution is performed on the reduced dimension feature image, the number of model parameters may be significantly reduced, and this may lead to increased efficiency.

### 2.3 Process of DL automatic segmentation

DL‐based methods require an initial training stage during which the neural network is provided with a large number of labeled 3D images. The dCTV1 and dCTV2 models were, respectively, trained and validated in the training cohort (*n* = 157) and the validation cohort (*n* = 20) from the definitive radiotherapy datasets. Similarly, the pCTV1 model was, respectively, trained and validated in the training cohort (*n* = 272) and the validation cohort (*n* = 30) from the postoperative radiotherapy datasets. During the network training process, we applied the multi‐scale strategy with a 3D network, by which we first trained a coarse‐scale network for rapid positioning of target area and then a fine‐scale segmentation model for precisely delineating targets’ contours based on previous coarse‐scale network output. In pre‐processing, global normalization was used. We chose window level 40 and window width 700. The minimum and maximum CT values are ‐310 and 390, respectively. CT values between them are linearly normalized into the range [−1, 1]. CT values less than the minimum are set to ‐1 and those greater than the maximum are set to +1. For coarse model training, the images are resampled to [5 mm, 5 mm, 5 mm]. During fine model training, the images are resampled to [1 mm, 1 mm, 1 mm]. No data augmentation was applied. During post‐processing, the maximum connected domain was extracted for dCTV1 and pCTV1, while the connected domains larger than 5 cm^3^ were extracted for dCTV2. The learning rate is 1e^–4^, batch size is 6, patch size is [96, 96, 96], and the optimizer is Adam. The training hardware is Intel Xeon E5‐2683 v3 with 64 GB memory and 4 NVIDIA Titan Xp. For the definitive radiotherapy group, we trained 3000 epochs for 50 h. For the postoperative radiotherapy group, we trained 3000 epochs for 86 h. The predicting time is less than 1 s for one case.

### 2.4 Quantitative evaluation of algorithm accuracy

For the radical radiotherapy datasets, the dCTV1 and dCTV2 models constructed by the DL‐based algorithm in the training cohort were applied to the testing cohort. For the postoperative radiotherapy datasets, the pCTV1 model was applied to the corresponding testing cohort. According to previous studies, the segmentation results were evaluated by the DSC that measures the target overlap between the DL‐based auto‐segmentations and the manual contours,^24^ the MSD that measures the MSD between two contours (mm),^25^ and the HD and HD 95% that calculates the largest distance between two contour surfaces (mm).^26^

### 2.5 Clinical evaluation of DL‐based auto‐segmentation

ROs assessed the results from the evaluation group. Nine ROs were classified as junior, intermediate, or senior according to their qualifications. The interobserver variation was calculated by DSC and MSD between different ROs. Notably, the senior ROs in the evaluation group were not the senior ROs who generated the reference contours. The assessment included three aspects:

1. RO assessment: the clinical applicability of DL‐based auto‐segmentation was graded according to four levels, defined below:
  - Grade 1: The segmentation result does not need to be modified and can be used in clinical practice.
  - Grade 2: The algorithm can be used as an auxiliary contouring tool, since the segmentation result can be used in clinical practice after minor modifications.
  - Grade 3: The algorithm can be used as an auxiliary contouring tool, and the segmentation result can be used in clinical practice after significant modifications.
  - Grade 4: The algorithm has no auxiliary contouring value. In addition, perceived errors in the segmentation results have been identified.
2. Comparison of the DL‐based auto‐segmentation results with the contours by the ROs: images for four patients randomly selected from the testing group of definitive radiotherapy and six patients randomly selected from the testing group of postoperative radiotherapy were distributed to the nine ROs for manual contouring. In addition, the DL‐based auto‐segmentations were edited blindly by these ROs. The DSC, MSD, and HD were calculated to assess the contouring accuracy and variations.
3. Evaluation of time consumption: times spent on manual, only DL‐based automatic, and DL‐assisted contouring were recorded for efficiency comparison.

### 2.6 Statistical analysis

The paired *t*‐test was used to compare the DSC, MSD, and HD values between different models. The data are presented with mean ± standard deviation. All analyses were performed using SPSS statistical software (IBM SPSS, version 20.0; New York, NY, USA). Statistical significance was determined by a two‐tailed *p*‐value < 0.05.

The years of experience of the nine ROs are as follows:

1. Junior RO: Chang Cai (2 years), Jing Zhao (4 years), Fei Sun (6 years).

2. Intermediate RO: Wei Gong (8 years), Yi‐Ming Yao (9 years), Yuan Xu (14 years).

3. Senior RO: Qi Zhao (13 years), Li‐Li Wang (21 years), Xiao‐Ting Xu (22 years).

## 3 RESULTS

### 3.1 Performance of DL‐based auto‐segmentation

**FIGURE 3:**
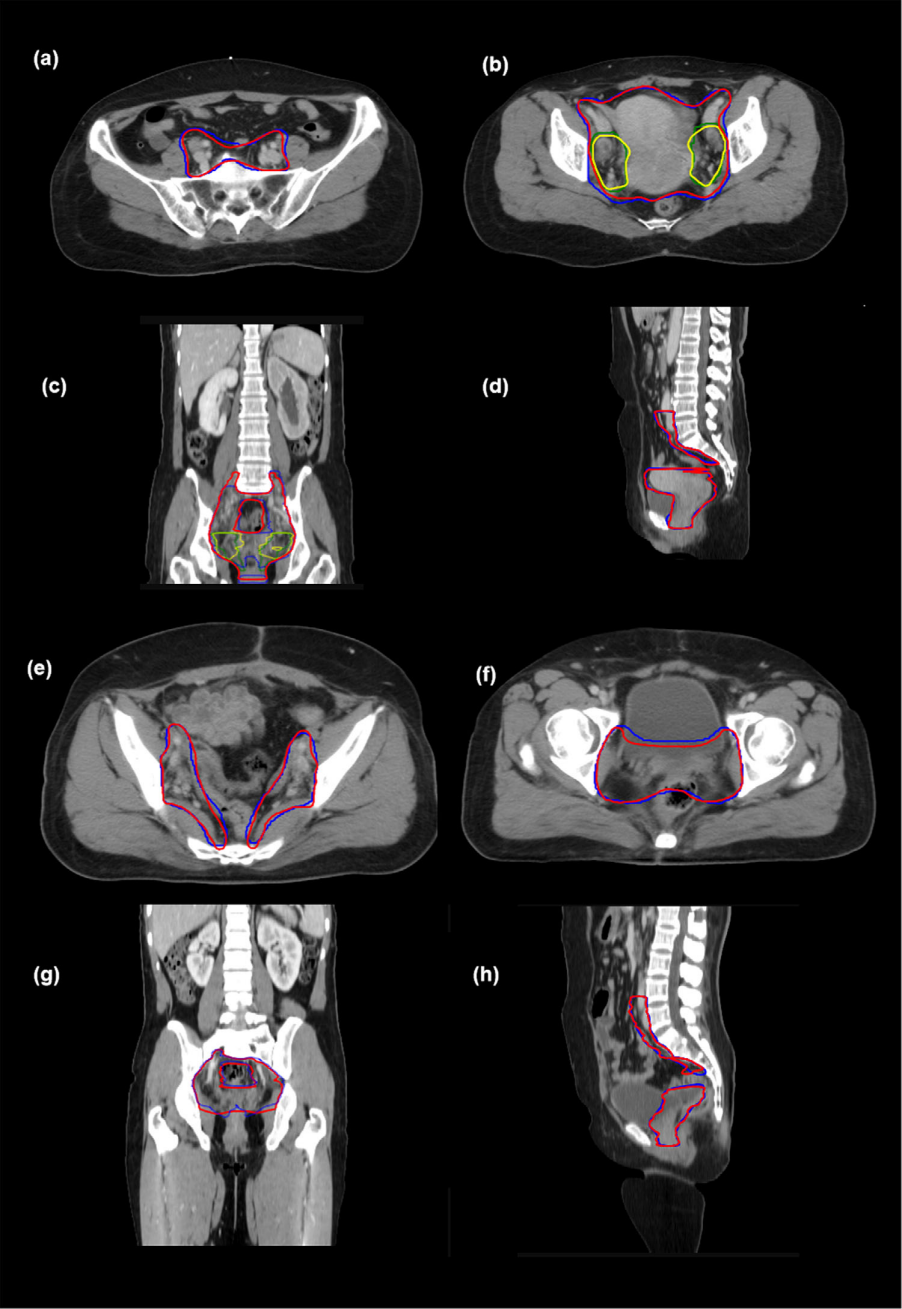
Comparison of the results between automatic segmentations and reference contours. (a and b) clinical tumor volume for definitive radiotherapy (dCTV)1 and dCTV2 in different cross‐sections, (c) coronal view, and (d) sagittal view. dCTV1 and dCTV2 of the reference are in red and yellow, respectively. dCTV1 and dCTV2 of the automatic segmentation are in blue and green, respectively; (e and f) clinical tumor volume for postoperative radiotherapy (pCTV)1 in different cross‐sections, (g) coronal view, and (h) sagittal view. pCTV1s of the reference and automatic segmentation are in red and blue, respectively

As for the test cases, the results for the accuracy of the DL‐based auto‐segmentations for dCTV1, dCTV2, and pCTV1 are presented in Table 1. As shown in Figure 3, one case with definitive radiotherapy and one case with postoperative radiotherapy were randomly selected from the corresponding testing groups for assessment of the level of concordance for the CTVs between the DL‐based auto‐segmentations and the reference contours. We observed DSCs of 0.88 ± 0.03, 0.70 ± 0.09, and 0.86 ± 0.03 for the dCTV1, dCTV2, and pCTV1, respectively. The MSDs for the dCTV1, dCTV2, and pCTV1 contours were 1.32 ± 0.48, 2.42 ± 1.62, and 1.15 ± 0.38 mm, respectively. All values were less than the accepted 3–5 mm margin of systematic and random error for radiation therapy for cervical cancer. The HDs for the dCTV1, dCTV2, and pCTV1 contours were 21.60 ± 7.50, 22.44 ± 8.49, and 20.78 ± 6.22 mm, respectively. These results indicate strong consistency between the DL‐based auto‐segmentation and the reference contours by senior ROs.

**TABLE 1:** Performance of deep learning (DL)‐based auto‐segmentation models (compared with the reference contours)

DL‐based models for different targets	DSC	MSD (mm)	HD (mm)	HD 95 (mm)
dCTV1	0.88 ± 0.03	1.32 ± 0.48	21.60 ± 7.50	4.86 ± 0.56
dCTV2	0.70 ± 0.09	2.42 ± 1.62	22.44 ± 8.49	6.47 ± 1.92
pCTV1	0.86 ± 0.03	1.15 ± 0.38	20.78 ± 6.22	4.11 ± 0.65

Abbreviations: dCTV, clinical tumor volume for definitive radiotherapy; DSC, dice similarity coefficient; HD, Hausdorff distance; HD 95, Hausdorff distance 95%; MSD, mean surface distance; pCTV, clinical tumor volume for postoperative radiotherapy.

Three cases in the test sets were randomly selected for contouring dCTV1, dCTV2, and pCTV1 to detect the limitation of the DL‐based algorithm, acquiring DSCs of 0.796, 0.435, and 0.807, respectively. The differences between the reference and the DL‐based contours mainly exist in superior and inferior boundaries, small intestine, rectum, and bladder.

### 3.2 Evaluation of the clinical value of DL‐based auto‐segmentation

According to the grading standard for contour accuracy described above, 2.4%, 63.5%, and 34.1% of the DL‐based auto‐segmentations were scored as grades 1, 2, and 3, respectively. These results indicated that most segmentations still needed to be modified in order to be considered clinically acceptable. However, the majority of them (63.5%) required only minor modifications. The main deficiencies of the auto‐segmentations were classified as inaccuracies in the top and bottom boundaries, contouring range, vascular expansion distance, and the muscle rectum compared with the reference contours.

**FIGURE 4:**
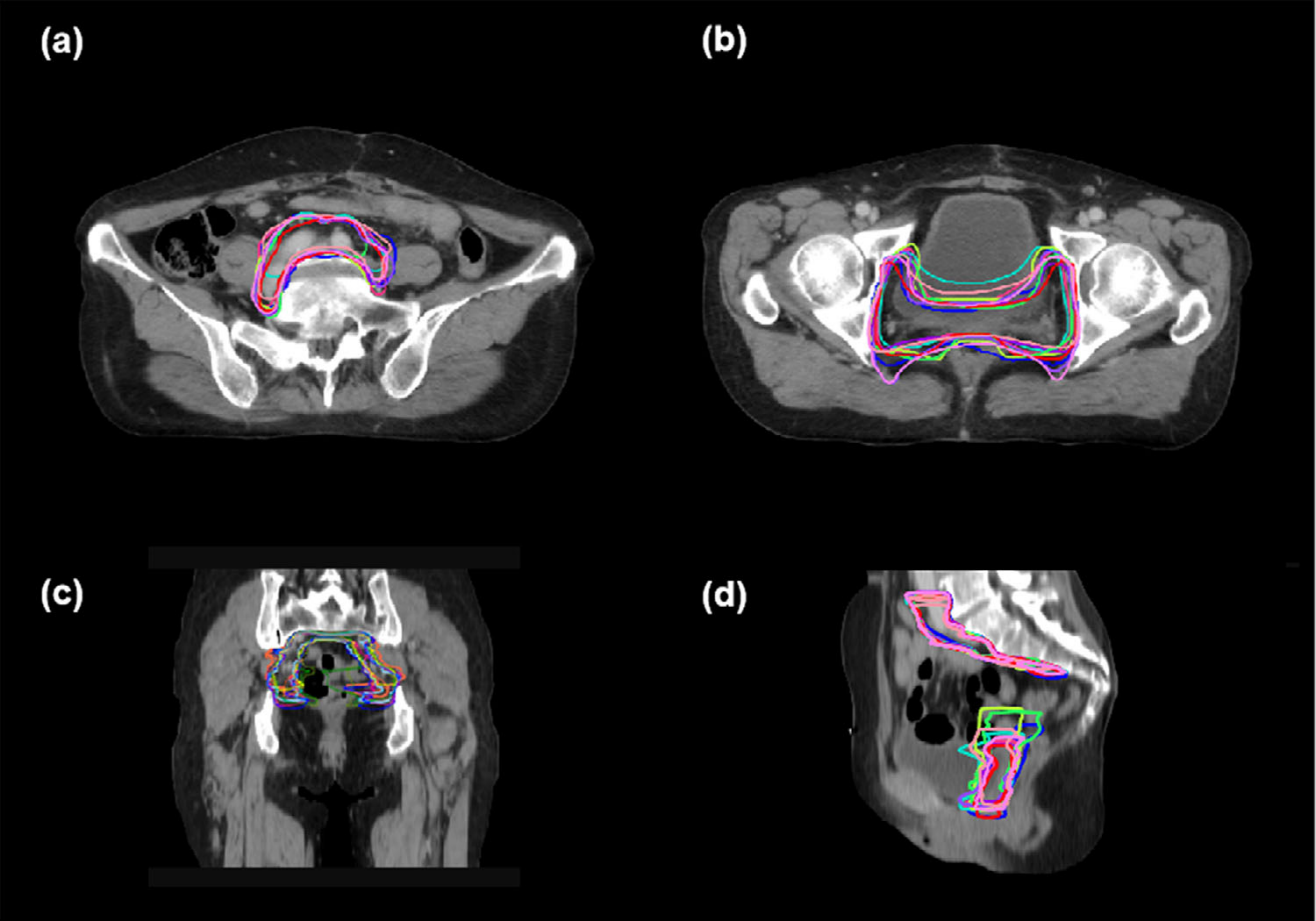
Comparison the results of manual contouring with automatic segmentation, in terms of the distribution of dice similarity coefficient (DSC), mean surface distance (MSD), and Hausdorff distance (HD) (*p < 0.05). Red boxes represent variations between deep learning (DL)‐based auto‐segmentations and the reference contours; green/dark blue/light blue boxes represent variations between the junior/intermediate/senior and the reference contours

To evaluate the clinical application value of the DL‐based auto‐segmentation, we compared the DL‐based tool with nine qualified ROs with different levels of qualification (junior, intermediate, and senior) in Figure 4. For dCTV1, DL‐based auto‐segmentation achieved contouring results comparable to the manual contours from ROs, as shown by the similar values for DSC, MSD, and HD. For dCTV2, the DL‐based tool outperformed the junior ROs as shown by a higher DSC value, was found to be superior to junior and intermediate ROs in terms of MDS, and performed better than all ROs in terms of HD. For pCTV1, the DL‐based tool was superior to junior ROs in terms of MSD and superior to intermediate ROs in terms of HD. Additionally, we observed a similar performance of the DL‐based tool to manual contouring by senior ROs in terms of DSC, MSD, and HD.

**FIGURE 5:**
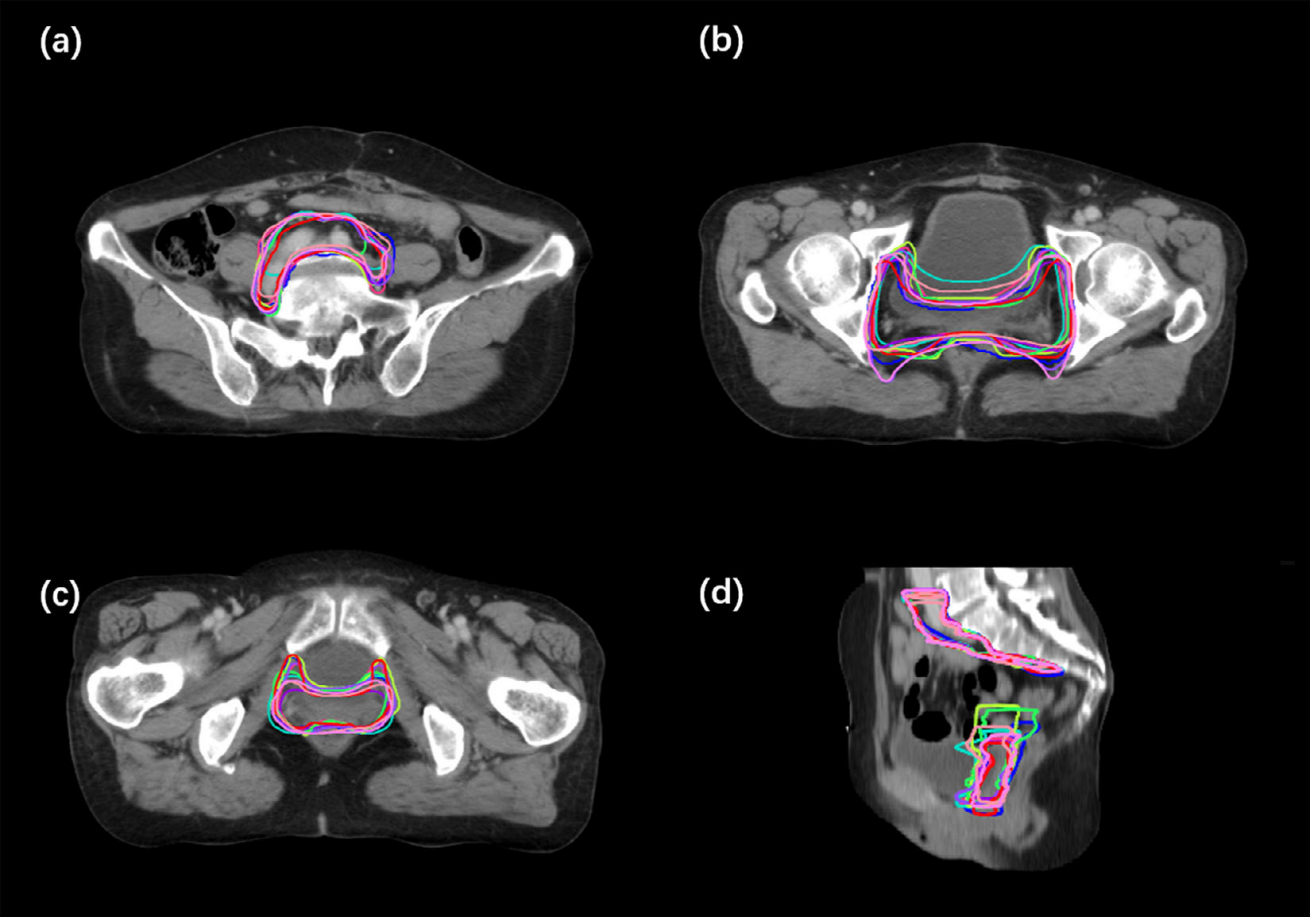
A median case with the reference, deep learning (DL) and all radiation oncologists (ROs) contours. (a and b) Three cross‐sections, (c) coronal view, and (d) sagittal view. Reference clinical target volumes (CTVs), DL contours, and all ROs contours are in red, blue, and other colors, respectively

Next, we conducted a comparison between manual contours and DL‐assisted manual contours from junior ROs to see whether the DL assistance could enhance the accuracy of manual contouring (shown in Figure 5). As shown in Table 2, DL assistance achieved higher DSC values both for dCTV2 and pCTV1 contouring (both *p*‐values < 0.05). Table 3 shows the interobserver variation between ROs. We calculated DSC and MSD between different ROs and the reference contour. The variation of each contour shows the interobserver variation.

**TABLE 2:** Dice similarity coefficients (DSCs) of contours provided by unassisted junior radiation oncologists (ROs) or deep learning (DL)‐assisted junior ROs

	DSC		
Models	Unassisted junior ROs	DL‐assisted junior ROs	*p*
dCTV2	0.57 ± 0.11	0.72 ± 0.08	<0.05
pCTV1	0.82 ± 0.03	0.85 ± 0.04	<0.05

Abbreviations: dCTV, clinical tumor volume for definitive radiotherapy; pCTV, clinical tumor volume for postoperative radiotherapy.

**TABLE 3:** Interobserver variation of radiation oncologists (ROs) and the comparison with deep learning (DL) results

		dCTV2				pCTV1					
		Case 1	Case 2	Case 3	Case 4	Case 1	Case 2	Case 3	Case 4	Case 5	Case 6
DSC	DL	0.767	0.686	0.732	0.837	0.830	0.867	0.826	0.871	0.885	0.870
	RO1	0.474	0.474	0.498	0.529	0.811	0.840	0.794	0.867	0.858	0.840
	RO2	0.525	0.429	0.491	0.522	0.746	0.815	0.768	0.853	0.873	0.846
	RO3	0.695	0.741	0.662	0.751	0.765	0.834	0.812	0.821	0.820	0.824
MSD (mm)	DL	1.586	2.878	1.867	0.934	0.928	1.008	1.487	1.110	0.833	1.039
	RO1	3.982	4.632	3.019	3.592	1.090	1.180	1.392	1.080	0.959	1.263
	RO2	5.520	7.127	6.001	4.286	1.523	1.416	1.472	1.074	0.854	1.115
	RO3	1.792	1.805	2.190	1.411	1.404	1.231	1.502	1.366	1.301	1.396

Abbreviations: dCTV, clinical tumor volume for definitive radiotherapy; DSC, dice similarity coefficient; MSD, mean surface distance; pCTV, clinical tumor volume for postoperative radiotherapy.

With regard to the contouring efficiency, times required for manual contouring by ROs with different qualifications and for DL‐assisted contouring were recorded and further compared in Table 4. Our data revealed that the DL‐based tool significantly reduced the average time spent on the contouring, taking less than 1 s versus 9–48 min for manual contouring by ROs, for dCTV1, dCTV2, and pCTV1. Specifically, for junior ROs after DL assistance, the average contouring time was reduced from 19.9 to 10.1 min (49.2%) for dCTV2 contouring and from 43.6 to 14.7 min (66.2%) for pCTV1 contouring.

**TABLE 4:** Average time requirement for deep learning (DL)‐based auto‐segmentation and manual contouring by radiation oncologists (ROs) with different qualifications

	Time (mean ± SD)		
Targets	dCTV1	dCTV2	pCTV1
Auto‐segmentation	0.8 ± 0.102 s	0.43 ± 0.083 s	0.93 ± 0.117 s
Junior ROs	48 ± 4.56 min	14 ± 6.94 min	44 ± 12.70 min
Intermediate ROs	31 ± 11.61 min	9 ± 1.42 min	36 ± 8.07 min
Senior ROs	26 ± 7.25 min	14 ± 4.88 min	20±1.82 min

Abbreviations: dCTV, clinical tumor volume for definitive radiotherapy; pCTV, clinical tumor volume for postoperative radiotherapy; SD, standard deviation.

## 4 DISCUSSION

The typical CTVs for cervical cancer radiotherapy planning are usually large. CTV position and shape are greatly affected by the filled state of the bladder, rectum, and other adjacent organs, which poses a challenge for the training of DL‐based auto‐segmentation models.^27^ In the current study, we evaluated the clinical value of DL‐based auto‐segmentation for CTV (dCTV1, dCTV2, and pCTV1) contouring of cervical cancer. We demonstrated that the DL tool achieved contouring results comparable to those of senior ROs and outperformed junior and intermediate ROs in the contouring of dCTV2 and pCTV1. In addition, the contouring accuracy of junior ROs was enhanced after initial contours were generated with DL assistance. Furthermore, the DL‐based auto‐segmentation greatly reduced the time required to delineate the CTVs. Our study confirmed the promising capability of DL‐based auto‐segmentation in delineating CTVs for cervical cancer.

The DL‐based auto‐segmentation performed well, achieving high DSC and low MSD values. However, the HD values could not be restrained to a low level (mean values > 20 mm). In view of the high HD values observed in this study, a portion of the results were selected to compare the differences between the auto‐segmentation and the reference contours. The inconsistencies were generally located around the small lymph nodes at the level of the femoral head. During the manual contouring process, ROs must refer to the diagnosis information to determine whether these targets should be included, whereas auto‐segmentation cannot distinguish them. Performance in this regard may be improved by: (1) increasing the diversity of the training data (with and without potential positive lymph nodes) and (2) improving the consistency of the training data (e.g., all lymph nodes are included or none are included). Meng et al.^28^ improved the HD value of automatic segmentation results via a method of post‐processing. In their study, the HD value for automatic liver segmentation decreased from 89.2 to 29.2 mm, and the HD value for automatic liver cancer segmentation decreased from 65.4 to 7.7 mm. In another report, the direct HD value was used to represent the maximum difference between two contours, which is very sensitive to abnormal contouring.^29^ For the automatic segmentation of CTV, post‐DL manual contouring and confirmation are generally needed to modify some abnormal points. In some studies,^30^, ^31^ 95% HD was used to evaluate the accuracy of automatic segmentation, and the results were on the order of several millimeters, indicating that 95% HD value may be a more suitable parameter for clinical evaluation of automatic segmentation results.

For the evaluation of the clinical value of DL‐based auto‐segmentation, the consistency between the automatic segmentation results and the manual contours provided by ROs was compared. We detected no significant differences between these contouring results for dCTV1; that is to say, the DL tool performed comparably to ROs with all qualifications for contouring dCTV1. However, for dCTV2 and pCTV1, the automatic segmentation results were roughly similar to the manual contours provided by senior ROs, but better than those provided the junior and intermediate ROs. In the current clinical workflow, radiotherapy planning is usually manually contoured by junior and intermediate ROs first, whereafter the contours are reviewed and modified by senior ROs. Thus, improving the target contouring skills of junior ROs in a comprehensive, systematic, and effective way is a key goal for achieving standardized radiotherapy training. Our results suggest that the DL tool can be used by junior and intermediate ROs to improve the consistency and accuracy of their contouring, so that the time spent by senior ROs on modifying the contours can be reduced.

The proficiency analysis showed that the times required for the manual contouring of CTVs ranged from 9 to 48 min in one case, shown in Table 2. In comparison, the DL‐based automatic segmentation method required not even 1 s. It is obvious that the automatic segmentation algorithm has a significant advantage over manual contouring. As we known, the complexity of targets and the experience of ROs determines the duration of the manual contouring process. If the DL‐based automatic segmentation model could be used as an assistance tool, the time required for the contouring of target volumes will be significantly reduced. However, the number of the evaluation cases is relatively small in our study. Record and compare the times required for manual contouring by ROs with different qualifications and for DL‐assisted contouring in cohorts with more evaluation cases is warranted.

The DL‐based auto‐segmentation appears to be well‐suited for CTV contouring for cervical cancer, because the large CTVs usually span many CT slices, each of which would otherwise need to be manually contoured. Additionally, we found that only minor modifications were needed for more than half of auto‐segmentations (63.5%) and significant modifications were needed for 34.1% of auto‐segmentations to meet the clinical requirements. This is mainly because the automatic segmentation algorithm is currently not capable of following some known fixed rules related to specific boundaries. One possible solution is to include as many of the identified normal tissues and boundaries as possible in the training data, so that the neural network is able to learn more anatomic spatial relationships. Alternatively, a hybrid algorithm that combines DL with logical target area contouring rules can be developed.

Our results showed that the DL‐based tool performed worse at the superior and inferior boundaries. We used a 3D DL model and demonstrated that the contouring would tend to be a smooth 3D structure when the CTVs suddenly appeared or disappeared in ground truth, and our algorithm should be optimized to solve this issue. No final conclusion has been reached on the other three items (intestine, rectum, and bladder). The output of the algorithm tends to follow a clear and definite rule, which infers some deviations in the consistency of the contours. This is the motivation for using an algorithm to improve the consistency.

Our study has several limitations that should be noted. Firstly, the current approach was not evaluated by an external test set. In theory, our model may work for other clinical centers which follow the same guidelines of target volume delineation. Secondly, we did not include dosimetric assessment for auto‐contouring evaluation, which is another important part in radiotherapy. We suggest ROs use this model to generate CTV contours, then review and correct these contours according to the clinical situation. After that, ROs can generate planning target volume by adding margins to CTV as usual, and the radiotherapy plans can be generated manually or automatically according to the confirmed contours.

## 5 CONCLUSIONS

In summary, our study verified the feasibility of the DL‐based automatic segmentation of CTVs for cervical cancer. We showed that a DL tool achieved comparable contouring accuracy to manual contouring by senior ROs and was superior to that provided by junior and intermediate ROs. Additionally, DL assistance can effectively enhance the contouring accuracy by junior ROs. Furthermore, the contouring time required was significantly reduced with the DL assistance for all ROs. Hence, the DL tool may serve as a promising method for improving the therapeutic effects of radiation for cervical cancer.

## AUTHOR CONTRIBUTIONS

Chen‐Ying Ma and Ju‐Ying Zhou conceived the idea of the study; Miao‐Fei Han, Yao‐Zong Gao, and Hui Du analyzed the data; Xiao‐Ting Xu and Jian Guo interpreted the results; Chen‐Ying Ma wrote the paper; all authors discussed the results and revised the manuscript.

## CONFLICT OF INTEREST

The authors declare no conflict of interest.

## ETHICAL APPROVAL

This study was approved by the ethics committee of Medical Ethic Committee of 1st Affiliated Hospital of Soochow University. All procedures performed in studies involving human participants were in accordance with the ethical standards of the institutional and/or national research committee and with the 1964 Helsinki declaration and its later amendments or comparable ethical standards.

## FUNDING INFORMATION

National Natural Science Foundation of China, Grant Number: 81602792; Suzhou Science and Technology Development Plan Project, Grant Number: SS201628.

## Supporting information

SUPPORTING INFORMATION

## Data Availability

The datasets generated and analyzed during the present study are available from the corresponding author on reasonable request.
