# Supplementary material for: Deep learning‐based auto‐segmentation of clinical target volumes for radiotherapy treatment of cervical cancer
Source: J Appl Clin Med Phys. 2021 Nov 22;23(2):e13470. doi: 10.1002/acm2.13470 (PMC8833283; doi:10.1002/acm2.13470)
Supplement: Supplementary file 1 — SUPPORTING INFORMATION [file ACM2-23-e13470-s001.doc]

**Deep learning-based auto-segmentation of clinical target volumes for radiotherapy treatment of cervical cancer**

Chen-ying Ma^1^, Ju-ying Zhou^1,*^, Xiao-ting Xu^1^, Jian Guo^1^, Miao-fei Han^2^, Yao-zong Gao^2^, Hui Du^2^, Johannes N. Stahl^2^, Jonathan S. Maltz^2^

^1^Department of Radiation Oncology, 1st Affiliated Hospital of Soochow University, Suzhou 215123, China

^2^Shanghai United Imaging Healthcare, Co. Ltd., Jiading 201807, China.

***Corresponding Author**

Ju-ying Zhou

Department of Radiation Oncology, 1st Affiliated Hospital of Soochow University, Suzhou 215123, China, No.188 Shizi Street

Tel: +8613962142066

Email: [zhoujuyingsy@163.com](mailto:zhoujuyingsy@163.com)

**Running title:** CTV auto-segmentation of cervical cancer

**Key words**: cervical cancer; clinical target volume (CTV); artificial intelligence (AI); deep learning; auto-segmentation.

**Author Contribution Statement**

Chen-ying Ma and Ju-ying Zhou conceived the idea of the study; Miao-fei Han, Yao-zong Gao and Hui Du analysed the data; Xiao-ting Xu and Jian Guo interpreted the results; Chen-ying Ma wrote the paper; all authors discussed the results and revised the manuscript.

**Acknowledgments**

Thanks are due to Li-Li Wang, Qi Zhao, Yuan Xu, Yi-Ming Yao, Wei Gong, Fei Sun, Jing Zhao, Chang Cai for their participation in the clinical evaluation of AI-based auto-segmentation.

**Conflict of Interest Statement**

Not applicable

**Ethical approval**

This study was approved by the ethics committee of Medical Ethic Committee of 1st Affiliated Hospital of Soochow University. All procedures performed in studies involving human participants were in accordance with the ethical standards of the institutional and/or national research committee and with the 1964 Helsinki declaration and its later amendments or comparable ethical standards.

**Funding**

This work was supported by National Natural Science Foundation of China (81602792); Suzhou science and Technology Development Plan Project (SS201628).

**Data availability**

The datasets generated and analyzed during the present study are available from the corresponding author on reasonable request.
